# Adult Mesenteric Lymphangioma Resulting in Small Bowel Obstruction

**DOI:** 10.7759/cureus.55090

**Published:** 2024-02-27

**Authors:** Timbre Backen, Matthew Hatch, Ashwin Kurian

**Affiliations:** 1 General Surgery, Hospital Corporation of America (HCA) Swedish Medical Center, Englewood, USA; 2 Minimally Invasive Surgery, Denver Esophageal and Stomach Center, Denver, USA

**Keywords:** acute care surgery, mesenteric mass, benign abdominal mass, mesenteric cystic lymphangioma, small bowel volvulus, general surgery, emergent general surgery, adult lymphangioma

## Abstract

A mesenteric cystic lymphangioma (MCL) is a rare condition that primarily manifests in children. This case report illustrates an unusual presentation of an MCL causing a small bowel obstruction with volvulus in an adult. We present a 31-year-old male who presented to our hospital with a small bowel obstruction. He underwent laparotomy, and a lymphatic mass acting as a lead point and causing small bowel volvulus was discovered intra-operatively. The patient underwent a small bowel with associated mass resection and primary anastomosis; he recovered well. The final pathology demonstrated an MCL. Despite the MCL being a rare entity in adults, it must be considered as the differential for various abdominal pathologies. Although the majority of these masses lack malignant potential, they should be resected, as they pose a risk of mechanical obstruction, torsion, and perforation. Prior descriptions include individual case reports of symptomatic lesions, proposed non-operative management, and follow-up imaging.

## Introduction

Mesenteric cystic lymphangiomas (MCLs) are abdominal masses postulated to develop from malformations within lymphatic vessels, resulting in the failure of lymphatic fluid drainage [1]. Although most mesenteric cysts are believed to be congenital anomalies, only 60% present during childhood [2]. This incidence of delayed presentation allows for additional theories concerning etiology, including prior trauma, surgery, inflammation, or radiation that may result in obstruction of lymphatic channels and eventual mass development [3]. The estimated incidence of MCLs ranges from 1 in 20,000 to 1 in 250,000 people [4]. Presentation in adulthood can be variable, ranging from incidental findings on imaging to mass effects on intra-abdominal or retroperitoneal organs to life-threatening obstructions and surgical emergencies [3,5,6]. Physical exam may reveal an abdominal mass in up to 60% of patients [1]. Abdominal CT scan with IV contrast is the gold standard for diagnosis; tumors typically appear homogenous and hypodense, with thin partitions and a fatty appearance of chylous fluid [7]. Treatment is excision or marsupialization, as there is a risk of life-threatening complications associated with an intrabdominal tethering point [8]. Pathologic evaluation is necessary to rule out the underlying malignancy. 

Our patient had been suffering from vague abdominal pains for years previously attributed to reflux or peptic ulcer disease without a definitive workup. On his presentation, he had an acute severe attack of abdominal pain, nausea, and vomiting and labs demonstrating leukocytosis. Imaging was consistent with an undefined tumor causing an acute transition point in his small bowel.

## Case presentation

**Figure 1:**
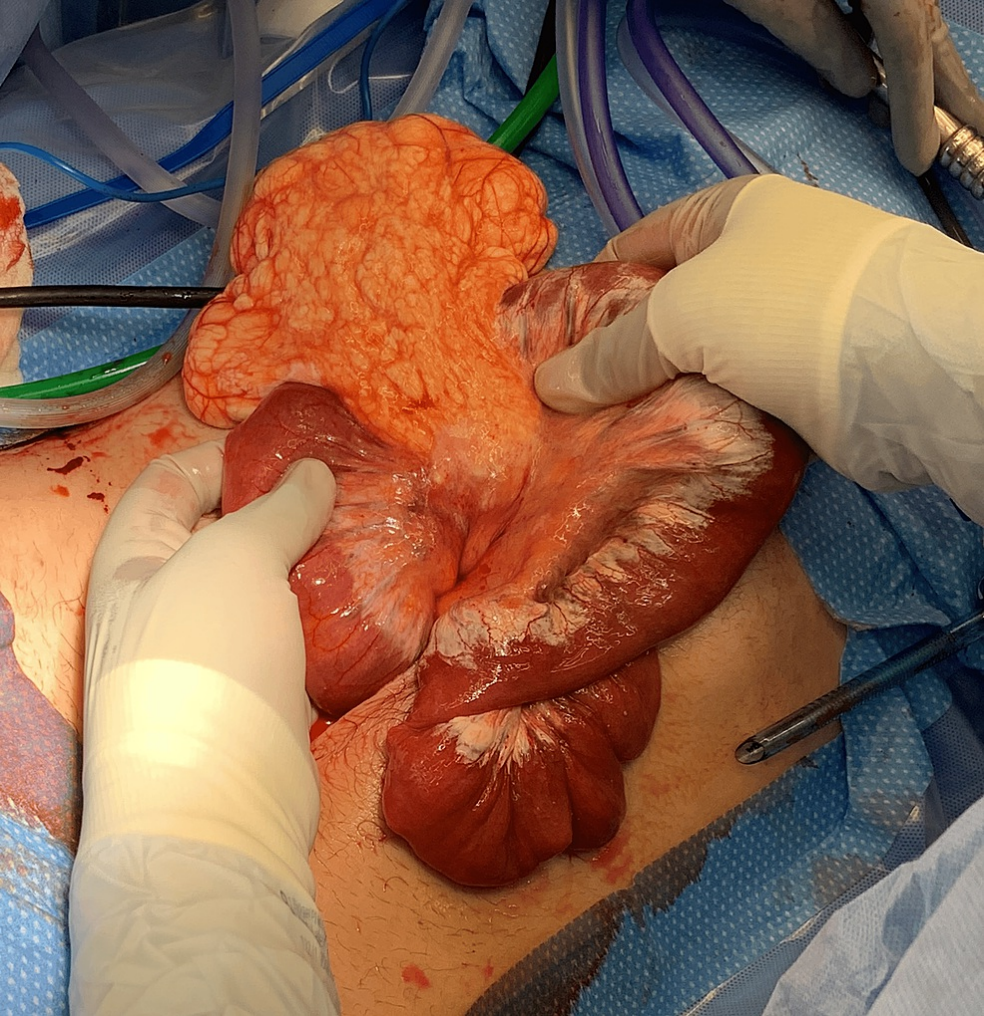
Mesenteric cystic mass with proximal small bowel lymphatic congestion

**Figure 2:**
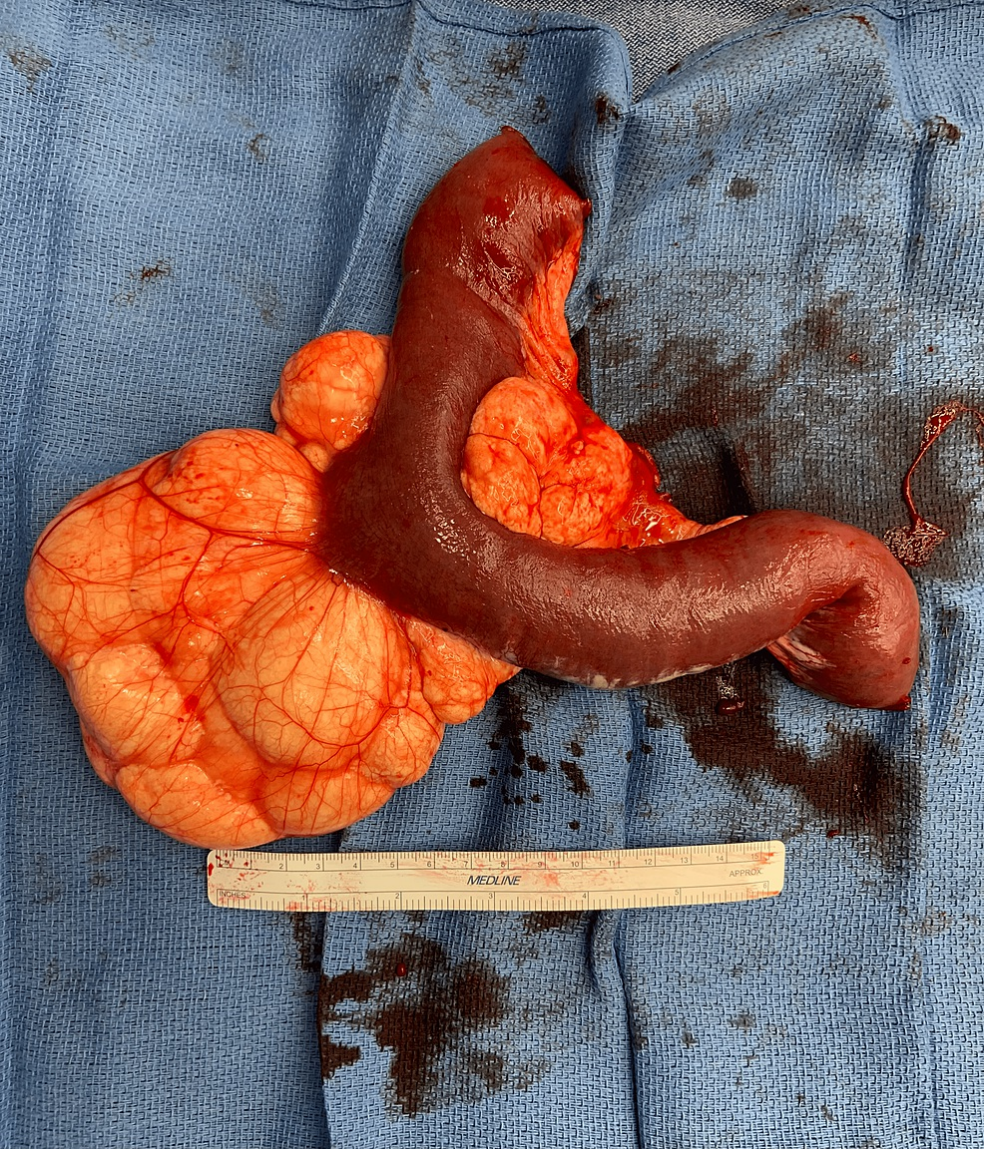
Resected small bowel with associated benign mesenteric cystic lymphangioma

Our patient is a 31-year-old male with no past surgical history who presented to urgent care with a chief complaint of epigastric pain that was severe and persistent over about 24 hours. He denied previous radiation or recent abdominal trauma. He did endorse associated nausea, persistent after two episodes of bilious emesis, as well as one episode of non-bloody diarrhea in the same time period. He endorsed an episode of similar pain years ago. He was prescribed famotidine and sucralfate for presumed peptic ulcer disease with marginal benefit, which the patient took for a few months and stopped independently. Otherwise, he had no significant medical history. At presentation, the patient was afebrile and hemodynamically stable, with no lactic acidosis and a mild leukocytosis of 12 x10^9/L (RR: 3.8-10.8 x10^9/L). A CT abdomen/pelvis scan with IV contrast demonstrated a moderate length of dilated small bowel centrally in the abdomen, measuring up to 3.2 cm in caliber. The small bowel proximal and distal remained decompressed with twisting of the mesentery, concerning a closed loop small bowel obstruction. Additionally, there was a 10-15 cm fluid density surrounding the mesentery of the dilated small bowel extending into the pelvis. The distribution of the fluid density was atypical, concerning a cystic mass. The patient was transferred to our institution that same morning, and we proceeded with a diagnostic laparoscopy that afternoon. Immediately upon abdominal entry, a dilated and hemorrhagic loop of the small bowel was encountered on the right side of the abdomen. There appeared to be a large mesenteric mass tethering the small bowel at the root of the mesentery, and we opted to convert to an open approach as visualization was difficult. After midline laparotomy, the entire small bowel was eviscerated. There was a large mesenteric cystic mass with fatty infiltrate and multiple septa at the mid-small bowel causing an internal volvulus and subsequent early tissue ischemia. Lymphatic congestion was visible along the proximal small bowel (Figures 1, 2).

The affected distal jejunal small bowel and mesentery were resected, totaling roughly 35 cm in length. We completed a side-to-side small bowel anastomosis with a linear GIA stapler and closed the mesenteric defect. The abdomen was washed out with normal saline, two channeled drains were placed, and the abdomen was closed. On post-operative day two, the patient experienced a significant drop in hemoglobin and two episodes of melena but did not require transfusion. A CT angiogram abdomen/pelvis was performed and did not demonstrate an active bleed. The patient was monitored closely and had an otherwise uncomplicated post-operative course. He was discharged appropriately on post-operative day six. This patient’s pathology results were significant for a benign MCL. There were negative resection margins, two benign lymph nodes, and no evidence of dysplasia or carcinoma. A CD31 immunohistochemical stain was performed and highlighted anastomosing lymphatic channels within the cystic mesenteric lesion. At the six-week follow-up, the patient continued to recover without concerns. He was tolerating a regular diet, bowel habits had returned to normal, and he had returned to work with minimal post-operative pain. There was no plan for further follow-up imaging or surgical follow-up unless new clinical indications arose.

## Discussion

This case of a benign MCL in an adult patient highlights several important clinical considerations. Abdominal cystic lymphangiomas make up fewer than 10% of all cystic lymphangiomas described [9]. They typically appear as multilocular cystic lesions with thin walls and homogenous serous content on CT [10]. The differential for acute small bowel obstruction remains broad and must include all growths that have the potential to cause mass effect or tether to surrounding structures; MRI is the gold standard imaging modality to exclude potential malignancy and follow-up unresected masses [10]. Our specimen stained positive for CD31, which is a vascular marker that labels both blood vessel and lymphatic endothelium [11]. As CD31 has low specificity for the identification of lymphangiomas, additional tissue markers such as D2-40, a transmembrane protein expressed by lymphatic endothelial cells, are frequently used during histologic exam. Of note, D2-40 is also expressed in seminomas and epithelioid-type mesothelial neoplasms, and this must be considered during diagnostic workup [11]. The Perrot Classification attempts to classify abdominal cystic masses based on their histopathologic findings [9]. The recommended treatment for MCLs is complete resection to a negative margin, which was fortunately achieved in our patient [9]. Historically, total resection has been recommended even for asymptomatic presentations of disease. Tan et al. proposed an algorithm for monitoring asymptomatic MCLs with repeat imaging for the first three to six months after diagnosis given reports of spontaneous partial regression of the mass [12]. Excluding patients who are poor surgical candidates, we continue to recommend excision. Negative margins are ideal, as recurrence rates range from 0% to 13.6%, most frequently seen in patients with partial excision, as well as those with retroperitoneal lymphangiomas [12]. If the tumor is confined to the mesentery without the involvement of the intestinal wall and the mesenteric artery supply can be dissected free, enucleation without bowel resection has been successful [13]. 

## Conclusions

In a patient with no surgical history, presentation concerning bowel obstruction, and a cystic or fatty appearing mass on imaging, the diagnosis of MCLs should be considered. Our patient underwent laparotomy with resection of the mesenteric mass and associated small bowel and anastomosis. He recovered well and was found to have negative margins on pathology. It is worth remembering that these rare tumors are almost universally benign but do pose a risk of mechanical obstruction, perforation, and torsion. We recommend resection of the mass with or without small bowel resection, provided that the mesenteric blood supply is preserved. 

## References

[1] (2019). Schwartz's Principles of Surgery. Schwartz's Principles of Surgery.

[2] Russell TA, Eilber FC (2019). Lesions of the omentum, mesentery, and retroperitoneum. Maingot's Abdominal Operations.

[3] Rajendran S, Hui TC, Lin NS (2023). Torsed mesenteric lymphangioma causing closed-loop small bowel obstruction in an adult patient. ANZ J Surg.

[4] Losanoff JE, Richman BW, El-Sherif A, Rider KD, Jones JW (2003). Mesenteric cystic lymphangioma. J Am Coll Surg.

[5] Azimi B, Bagherian Lemraski S, Kouchak Hosseini SP, Khoshnoudi H, Aghaei M, Haghbin Toutounchi A (2023). Small bowel volvulus and mesenteric ischemia induced by mesenteric cystic lymphangioma in an adult and literature review; a case report. Int J Surg Case Rep.

[6] Losanoff JE, Kjossev KT (2005). Mesenteric cystic lymphangioma: unusual cause of intra-abdominal catastrophe in an adult. Int J Clin Pract.

[7] Thiam O, Faye PM, Niasse A (2019). Cystic mesenteric lymphangioma: a case report. Int J Surg Case Rep.

[8] Rossini M, Annicchiarico A, De Giorgi F, Del Rio P, Viani L (2020). Symptomatic giant mesenteric cystic lymphangioma in adulthood. ACG Case Rep J.

[9] Aliukonis V, Lasinskas M, Pilvelis A, Gradauskas A (2021). Pathological discrepancy: simple mesenteric cyst vs. mesenteric lymphangioma. Case Rep Surg.

[10] Raufaste Tistet M, Ernst O, Lanchou M, Vermersch M, Lebert P (2020). Imaging features, complications and differential diagnoses of abdominal cystic lymphangiomas. Abdom Radiol (NY).

[11] Ellis CL, Banerjee P, Carney E, Sharma R, Netto GJ (2011). Adrenal lymphangioma: clinicopathologic and immunohistochemical characteristics of a rare lesion. Hum Pathol.

[12] Tan DT, Chok AY, Farah BL, Yan YY, Toh EL (2019). Spontaneous partial regression of a microcystic jejunal mesenteric lymphangioma and a proposed management algorithm. BMJ Case Rep.

[13] Chen CW, Hsu SD, Lin CH, Cheng MF, Yu JC (2005). Cystic lymphangioma of the jejunal mesentery in an adult: a case report. World J Gastroenterol.

